# Reassessing the Safety of Pill-in-the-Pocket Propafenone

**DOI:** 10.7759/cureus.46282

**Published:** 2023-09-30

**Authors:** Andrew G Kim, Majid Yavari, Rand Sabanci, Esosa Ukponmwan, Fatima Ali-Ahmed

**Affiliations:** 1 Internal Medicine, Michigan State University, East Lansing, USA; 2 Internal Medicine, Michigan State University, E.W. Sparrow Hospital, East Lansing, USA; 3 Cardiac Electrophysiology, Michigan State University, East Lansing, USA

**Keywords:** cardioversion, antiarrhythmic, atrial flutter, atrial fibrillation, propafenone

## Abstract

The current guidelines state that propafenone can be used in combination with a beta-blocker or a calcium channel blocker for pharmacologic cardioversion of recent-onset atrial fibrillation in patients without structural heart disease. To prevent the conversion from atrial fibrillation to atrial flutter with a rapid ventricular rate, it is recommended to administer propafenone following the administration of a beta-blocker or a calcium channel blocker. However, this combination carries the potential risk of cardiogenic shock. There are several scenarios where this combination can lead to shock, attributed to the variable pharmacokinetics of propafenone among individuals and its significant drug interactions with commonly used AV nodal blockers, such as metoprolol and diltiazem. Additionally, a significant proportion of the population has genetic polymorphisms that affect the metabolism of these medications. While pill-in-the-pocket propafenone is also employed in outpatient settings, unexpected severe and life-threatening reactions have been reported. In this context, we present a case report of severe propafenone toxicity in a closely monitored inpatient setting aimed at converting atrial fibrillation.

## Introduction

Propafenone is classified as a class IC antiarrhythmic medication, exerting its effects through sodium channel-blocking activity that extends the effective refractory period and raises the diastolic excitability threshold [1]. Additionally, propafenone exhibits structural similarities to beta-blockers and possesses both beta-adrenergic blocking activity and calcium channel blocking activity [2]. It is widely recognized as a pill-in-the-pocket medication due to its ability to effectively terminate paroxysmal episodes of atrial fibrillation [3].

If pharmacologic cardioversion has been shown to be effective and safe in a monitored setting, it can be utilized as a pill-in-the-pocket approach to terminate atrial fibrillation outside of the hospital. Before utilizing the pill-in-the-pocket approach in an unmonitored outpatient setting, it is recommended to conduct an initial conversion trial in a monitored setting. This is important because the termination of atrial fibrillation can sometimes lead to bradycardia caused by sinus node or AV node dysfunction, or it can trigger a proarrhythmic response [4].

Even with initial monitoring in an inpatient setting, there are still potential risks of complications even after promptly managing life-threatening side effects. Several genetic and metabolic factors can exacerbate the unpredictable dose response of propafenone, particularly when used in conjunction with other AV nodal-blocking agents. These factors contribute to the complexity and variability of the drug's effects, emphasizing the need for careful monitoring and individualized management when using propafenone.

## Case presentation

A 53-year-old African-American male, with a medical history of paroxysmal atrial fibrillation (CHA2DS2-VASc score of 2), hypertension, diastolic heart failure, and depression, presented to the emergency department with progressively worsening shortness of breath and palpitations over the past few days. He had not been taking his prescribed medications, which included diltiazem, losartan, chlorthalidone, and amlodipine, as he was unable to get refills at the pharmacy. However, he had been consistently taking rivaroxaban and sertraline.

**Figure 1:**
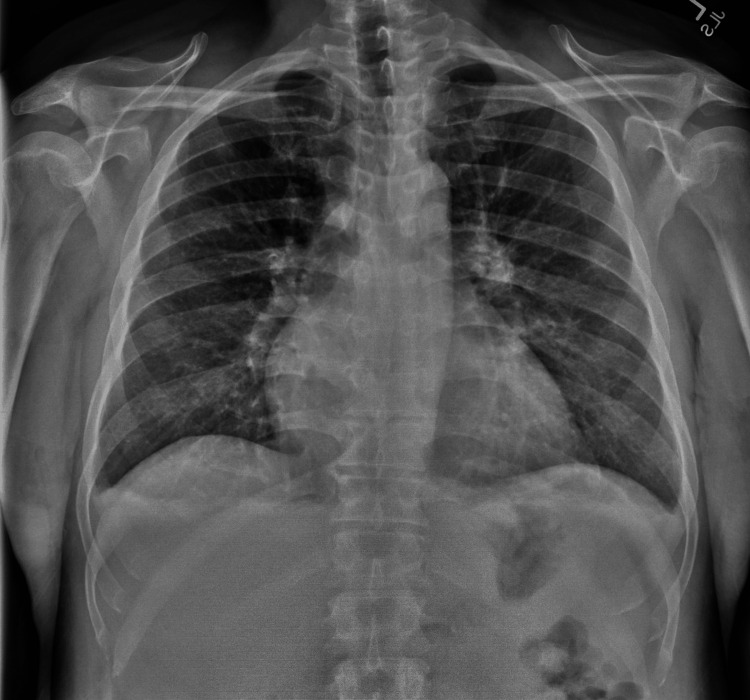
The chest X-ray upon arrival at the emergency department.

**Figure 2:**
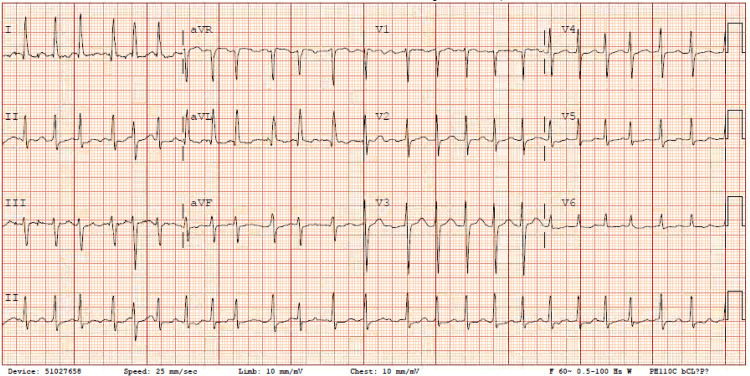
The initial ECG showing atrial fibrillation with a ventricular rate of 149 bpm.

The patient had a blood pressure of 154/82 mmHg and a heart rate of 152 bpm. Physical examination revealed an irregular heart rate and bilateral 2+ pretibial pitting edema. Chest X-ray (Figure 1) shows mild bilateral pleural effusion, and the electrocardiogram (ECG) indicated atrial fibrillation with a rapid ventricular response (Figure 2). Recent echocardiogram findings revealed a left ventricular ejection fraction of 65%, diastolic dysfunction, mild left ventricular hypertrophy, mild dilatation of the left atrium, and mild mitral regurgitation. To alleviate the patient's shortness of breath and fluid overload, he was administered IV furosemide 40 mg, and cardiac electrophysiology was consulted.

The exact onset of the current atrial fibrillation episode was uncertain, but it was suspected to be recent. He was diagnosed with paroxysmal atrial fibrillation six years ago, and his most recent office ECG, taken three months ago, showed sinus rhythm. Approximately four months ago, he experienced a documented episode of atrial fibrillation that spontaneously converted to sinus rhythm the following day. He is typically asymptomatic when experiencing atrial fibrillation.

To manage the symptomatic paroxysm of atrial fibrillation, we opted for a pill-in-the-pocket approach and provided the patient with a single oral dose of 600 mg propafenone. This decision was based on the patient's recent onset of atrial fibrillation without structural heart disease and his compliance with anticoagulation therapy. We initiated an IV diltiazem drip for rapid rate control and administered oral metoprolol extended release 50 mg before administering propafenone. The IV diltiazem drip was titrated to keep the patient's heart rate below 110 bpm. If the patient responded positively to this treatment approach during his hospitalization, our plan was to provide follow-up care as an outpatient.

**Figure 3:**
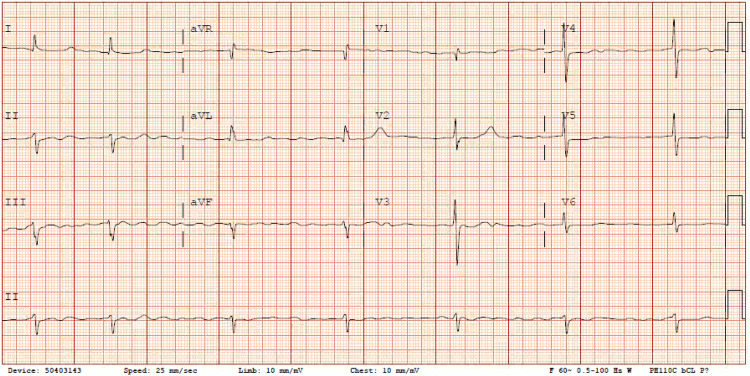
The ECG taken about two hours after the administration of propafenone showing atrial fibrillation with a ventricular rate of 41 bpm.

After one hour, the patient achieved a heart rate below 110 bpm, and the IV diltiazem drip was discontinued after administering a total of 10 mg. One hour later, the patient reported experiencing a headache and nausea, with normal vital signs showing a blood pressure of 103/84 mmHg and a heart rate of 98 bpm. Another hour later, the patient's mental status deteriorated to an obtunded state, accompanied by vital signs indicating a blood pressure of 81/45 mmHg and a heart rate as low as 24 bpm, while atrial fibrillation persisted. The ECG around that time revealed atrial fibrillation with a slow ventricular response, measuring 41 bpm (Figure 3).

Propafenone and all AV nodal blockers were discontinued, and the patient received an IV fluid bolus and IV atropine 1 mg. His symptoms slowly improved, and hemodynamic instability resolved. However, his relatively soft blood pressure and low normal heart rate persisted for up to 15 hours.

**Figure 4:**
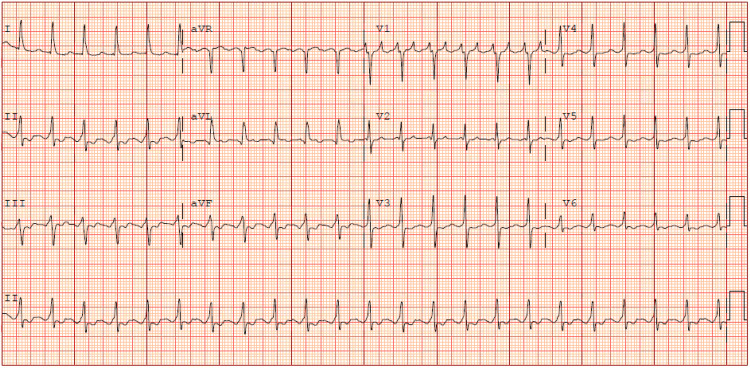
The ECG on the following day showing atrial flutter with a ventricular rate of 137 bpm.

The next day, a repeated laboratory analysis indicated evidence of multiorgan ischemia resulting from cardiogenic shock during the episode. The results showed a lactate level of 3.4 mg/dL, an increase in creatinine from an initial value of 1.0-2.1 mg/dL following the episode, a high-sensitivity troponin I level rising from 10 to 59 ng/L, and elevated AST levels from 73 to 262 U/L and ALT levels from 44 to 107 U/L (Table 1). Additionally, the patient experienced a recurrence of tachycardia, and the ECG revealed atrial flutter (Figure 4).

**Table 1:** Laboratory values prior to and following the event.

	Day 0	Day 1
Lactate (mg/dL)		3.4
Troponin I high sensitivity (ng/L)	10	59
Creatinine (mg/dL)	1	2.1
AST (U/L)	73	262
ALT (U/L)	44	107

**Figure 5:**
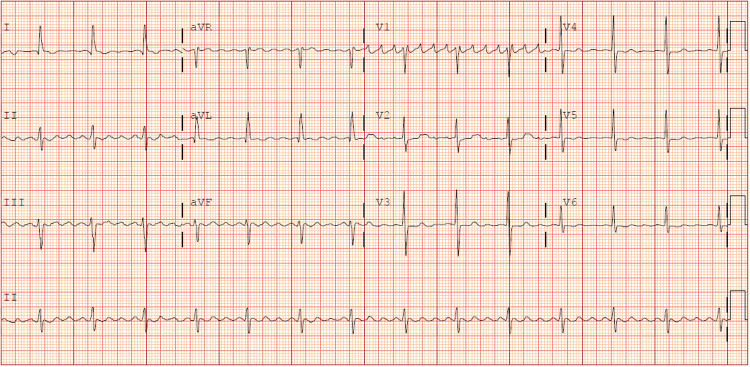
The ECG on discharge showing atrial flutter with a predominant 4:1 AV block at a ventricular rate of 83 bpm.

We presented the options of electrical cardioversion and a catheter ablation procedure, but the patient expressed reluctance to pursue further interventions. The patient consented only to taking diltiazem, a medication he had been using at home, without experiencing any adverse reactions. As a result, the patient was discharged with atrial flutter under control for ventricular rate (Figure 5).

Propafenone not only failed to chemically cardiovert the patient's atrial fibrillation with a rapid ventricular rate to normal sinus rhythm but also led to atrial fibrillation with a severely low ventricular rate, and later to atrial flutter.

On follow-up, the patient was rhythm-controlled with amiodarone. The rhythm has since been converted to normal sinus rhythm, and the patient is now considering a catheter ablation procedure. The patient was suggested to undergo CYP2D6 genetic testing; unfortunately, it could not be performed due to a non-medical reason.

## Discussion

Clinical dilemma of co-administering propafenone with other AV nodal blockers

According to the 2014 AHA/ACC/HRS guidelines for the management of atrial fibrillation, propafenone can be used in conjunction with a beta-blocker or a non-dihydropyridine calcium channel blocker in certain populations to convert atrial fibrillation to sinus rhythm [5]. The guidelines recommend that patients take an AV nodal-blocking agent, such as a beta-blocker or a non-dihydropyridine calcium channel blocker, prior to initiating propafenone as a pill-in-the-pocket approach. One reason for using a Vaughan Williams class IC agent, such as propafenone, at least 30 minutes after administering AV nodal-blocking agents in patients with atrial fibrillation is to prevent conversion to atrial flutter with 1:1 conduction, which can lead to rapid ventricular rates [6].

However, propafenone differentiates itself from other class IC antiarrhythmic medications due to its additional beta-adrenergic blocking activity and calcium channel blocking activity, although the latter is minimal at usual doses [2]. Additionally, propafenone demonstrates greater selectivity for cells with a high rate of activity but also exerts a stronger blocking effect on normal cells compared to class IA or IB antiarrhythmic drugs [7]. This property can potentially lead to excessive beta-blockade and calcium channel blockade when propafenone is combined with other AV nodal blockers. Despite the potential risks associated with additive and synergistic beta-blockade when propafenone is used in conjunction with other AV nodal blockers, co-administration is generally recommended due to the benefits it provides in most cases, especially for the conversion of atrial fibrillation with rapid ventricular response, as previously discussed [6].

Genetic polymorphism and highly variable pharmacokinetics among individuals

Propafenone is metabolized by CYP2D6, CYP3A4, and CYP1A4 enzymes [8]. CYP2D6 exhibits significant interindividual variability, resulting in variations in individual responses. Furthermore, studies have shown that propafenone demonstrates a significantly non-linear relationship between dose and serum concentration [9]. Approximately 6% of Caucasians are identified as poor metabolizers of propafenone due to a deficiency in CYP2D6 [8]. Other ethnic groups have a lower incidence of poor metabolism [10].

Although confirmatory genetic testing could not be performed, the severe clinical response suggests that the patient, as an African-American, may also be a poor metabolizer of CYP2D6 [10]. This case highlights the importance of caution regardless of ethnicity, especially when co-administering propafenone with AV nodal blockers or other cytochrome inhibitors.

Significant drug-drug interactions of propafenone

**Figure 6:**
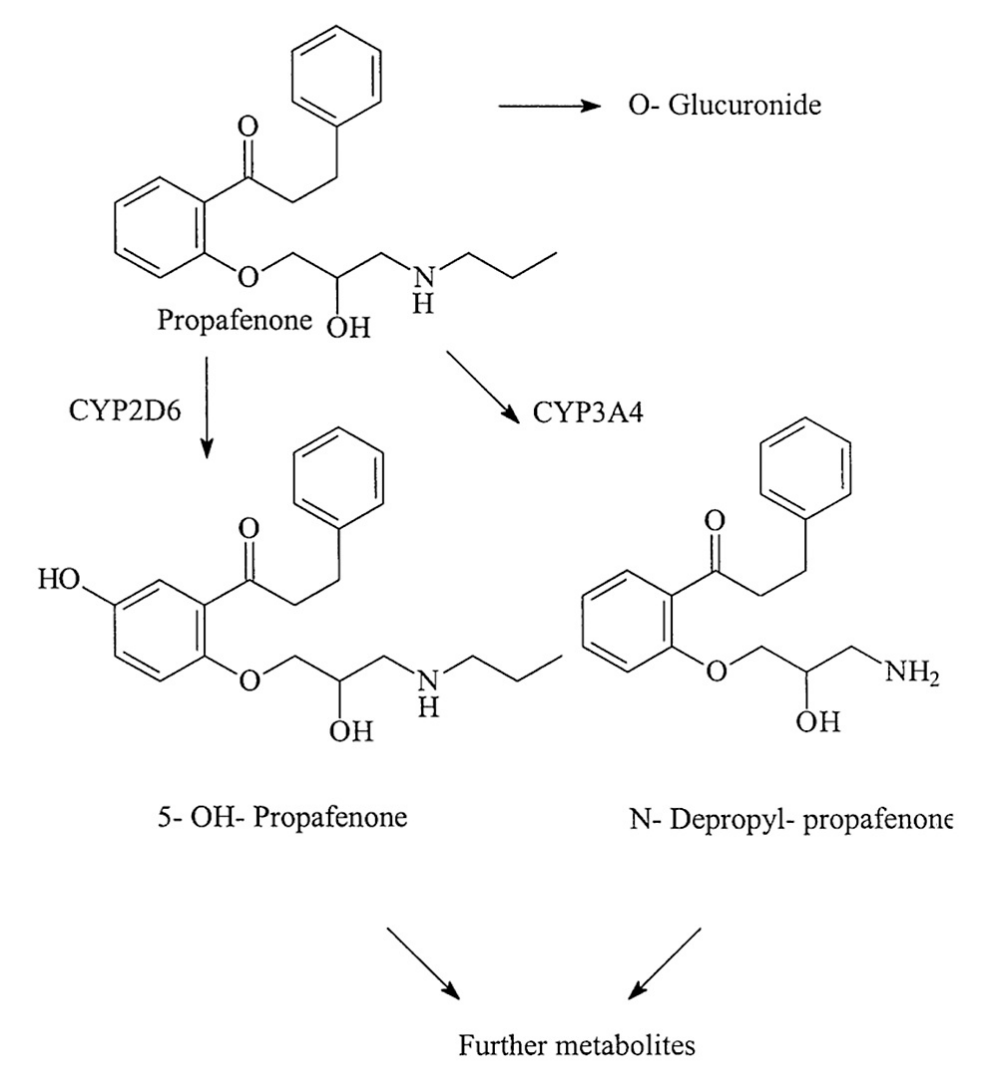
Metabolic pathway of propafenone. Propafenone is metabolized by cytochrome P450 enzymes, mainly 2D6 and 3A4 [11].

Moreover, if a patient takes medications that interact with CYP2D6 and CYP3A4, there is a higher likelihood that the serum concentration of propafenone will be outside the therapeutic range. Propafenone is primarily metabolized by CYP2D6 and CYP3A4 (Figure 6) [11]. CYP2D6 metabolizes approximately 20-25% of the currently available drugs [12], and CYP3A4 metabolizes approximately 50% of marketed drugs [13]. Metoprolol's metabolism occurs primarily via the CYP2D6 enzyme, with reported estimates of approximately 70-80% [14]. Additionally, diltiazem is subjected to extensive and highly variable hepatic first-pass metabolism by CYP3A4 [15]. It is worth noting that the patient was also taking sertraline for depression, which inhibits CYP2D6 [16]. Therefore, significant metabolic interactions with metoprolol and diltiazem, as well as shared beta-blockade activity and calcium channel-blocking activity with propafenone, have the potential to significantly potentiate beta-blockade and calcium channel blockade, especially in certain genetically predisposed populations.

## Conclusions

The current guideline recommends propafenone's use in combination with either a beta-blocker or a non-dihydropyridine calcium channel blocker for converting atrial fibrillation to sinus rhythm in specific patient populations. However, caution is necessary when combining propafenone with these medications due to the potential for unexpectedly severe reactions, especially when administering certain beta and calcium channel blockers that share metabolic pathways with propafenone in patients suspected to be poor metabolizers. This heightened risk is attributed to propafenone's activities involving both beta-blockade and calcium channel blockade, genetic variations in cytochrome metabolism, an unpredictable dose-response relationship, and the potential for significant drug-drug interactions.
